# Functional Exercise Capacity and Exploratory ICF‐Aligned Interpretation of the Incremental Shuttle Walk Test in Children With Asthma

**DOI:** 10.1002/ppul.71734

**Published:** 2026-07-27

**Authors:** Danilo Rufino Cavalcante de Souza, Ivan Peres Costa, Etiene Farah Teixeira de Carvalho, Dirceu Costa, Evelim Leal de Freitas Dantas Gomes

**Affiliations:** ^1^ Universidade de São Paulo – FMUSP São Paulo Brazil; ^2^ Universidade Nove de Julho‐ UNINOVE São Paulo Brazil

**Keywords:** activity limitation, functional exercise capacity, Incremental Shuttle Walk Test, International Classification of Functioning, pediatric asthma

## Abstract

**Background:**

Children with asthma may experience functional exercise limitations that are not fully reflected by resting lung function or conventional clinical assessments. Field‐based exercise tests provide complementary information regarding functional capacity, and the International Classification of Functioning, Disability and Health (ICF) offers a biopsychosocial framework to contextualize activity limitations. However, the integration of objective exercise performance with ICF‐aligned interpretation in pediatric asthma remains limited.

**Objective:**

To interpret functional exercise capacity in children with asthma using Incremental Shuttle Walk Test (ISWT) performance through an exploratory operational framework conceptually aligned with ICF activity qualifiers and to examine its association with lung function, asthma control, quality of life, and demographic characteristics.

**Methods:**

In this cross‐sectional study, 39 children with asthma (mean age 8.6 ± 2.3 years) underwent spirometry, completed the Asthma Control Questionnaire (ACQ‐6) and the Pediatric Asthma Quality of Life Questionnaire (PAQLQ), and performed the ISWT. Functional exercise capacity was expressed as a percentage of predicted ISWT distance and interpreted using an exploratory operational framework conceptually aligned with generic ICF activity qualifiers, generating two categories of activity limitation (mild/moderate and severe).

**Results:**

Resting lung function was relatively preserved (mean FEV_1_ 82.0 ± 16.4% predicted), whereas functional exercise capacity was markedly reduced (mean ISWT 41.4 ± 18.7% predicted). Based on the exploratory ICF‐aligned framework, 72% of participants were categorized as having severe activity limitation. Children with severe limitations walked significantly shorter distances than those with mild/moderate limitations (32.0 ± 11.5% vs. 65.3 ± 10.1% predicted; *p* < 0.001), with a very large effect size. Spirometric indices, asthma control, and quality of life scores did not differ between functional categories. ISWT performance was not correlated with lung function, asthma control, or quality of life and demonstrated a negative association with age.

**Conclusion:**

Children with asthma may present substantial functional exercise limitations despite relatively preserved lung function and similar levels of asthma control and quality of life. The exploratory operational alignment of ISWT performance with generic ICF activity qualifiers may provide a clinically useful framework to support interpretation of activity limitations not captured by conventional assessments. However, this approach should be interpreted cautiously and requires future validation before broader clinical application.

## 1 Introduction

Asthma is the most common chronic noncommunicable disease in childhood and remains a leading cause of morbidity worldwide, with significant impacts on daily activities, school attendance, and overall well‐being [1]. Although asthma is primarily characterized by chronic airway inflammation and variable airflow limitation, its clinical expression extends beyond respiratory symptoms, encompassing reduced physical activity, exercise intolerance, sleep disturbances, and impaired quality of life [2, 3, 4]. Current clinical management emphasizes symptom control and lung function assessed at rest; however, these measures may not fully reflect the functional consequences of the disease in everyday life [1, 5].

In pediatric asthma, resting spirometric indices such as forced expiratory volume in 1 s (FEV_1_) are often within normal or near‐normal ranges, particularly during periods of clinical stability [6]. Nevertheless, evidence indicates that children with asthma frequently present reduced exercise capacity and lower levels of physical fitness compared with healthy peers, even in the absence of significant airflow obstruction [7, 8, 9]. This dissociation between pulmonary function at rest and functional performance during physical exertion highlights an important gap in routine clinical assessment, as functional limitations may remain undetected when evaluation is restricted to traditional lung function tests [10].

Field‐based exercise tests offer a practical and clinically meaningful approach to evaluating functional exercise capacity in children with chronic respiratory diseases. The Incremental Shuttle Walk Test (ISWT) is an externally paced, incremental test that reflects the integrated responses of the respiratory, cardiovascular, and musculoskeletal systems [11]. The ISWT has demonstrated good validity and reproducibility in pediatric populations and allows exercise performance to be expressed as a percentage of predicted values based on age, sex, and anthropometric characteristics [12, 13]. Importantly, performance on field walking tests has shown limited association with resting spirometric parameters, reinforcing the need for complementary functional assessments in pediatric asthma [8, 10].

Beyond pulmonary function, several factors have been shown to influence functional capacity in children with asthma, including physical inactivity, sleep disturbances, airway inflammation, and disease control [5, 14, 15, 16]. Recent evidence indicates that poor sleep quality and sleep‐disordered breathing are associated with worse asthma control, increased pulmonary inflammation, reduced exercise capacity, and impaired quality of life in this population [16, 17]. These findings underscore the multidimensional nature of asthma‐related morbidity and the limitations of relying solely on physiological measurements at rest.

The International Classification of Functioning, Disability and Health (ICF) provides a comprehensive biopsychosocial framework for describing health and disability by integrating body functions, activities, participation, and contextual factors [18]. Within this framework, reduced exercise performance can be conceptualized as a limitation in the domain of activity. Although the ICF has been increasingly adopted in rehabilitation research, its application in pediatric asthma remains limited, particularly regarding the operational integration of objective exercise test results into clinically interpretable activity qualifiers [19]. Aligning functional exercise performance with ICF activity qualifiers may facilitate a more comprehensive understanding of functional impairment and improve interdisciplinary communication and clinical decision‐making.

Previous studies have applied the International Classification of Functioning, Disability and Health (ICF) as a conceptual framework to better understand the multidimensional impact of asthma in children. In this context, Gomes et al. [20] demonstrated that commonly used asthma‐specific quality of life instruments, such as the Pediatric Asthma Quality of Life Questionnaire (PAQLQ), predominantly capture domains related to body functions, with limited representation of activity and participation components when linked to the ICF framework. These findings highlight an important gap in the functional assessment of pediatric asthma, as subjective questionnaires may fail to adequately reflect activity limitations experienced during daily physical exertion. Therefore, incorporating objective functional measures aligned with the ICF may provide complementary and clinically meaningful information on activity limitations that are not fully captured by conventional clinical or patient‐reported outcomes.

Therefore, the aim of this study was to classify functional exercise capacity in children with asthma using ISWT performance expressed as a percentage of predicted values and operationally aligned with ICF activity qualifiers. Additionally, we sought to examine the association between functional exercise capacity and lung function, asthma control, quality of life, and demographic characteristics. We hypothesized that functional exercise limitation identified through an ISWT–ICF classification would be largely independent of resting lung function and clinical control, thereby revealing a dimension of asthma‐related impairment not captured by conventional clinical assessments.

## 2 Methods

### 2.1 Study Design and Ethical Considerations

This was a cross‐sectional observational study conducted in accordance with the Declaration of Helsinki and reported in accordance with the Strengthening the Reporting of Observational Studies in Epidemiology (STROBE) guidelines. The study was approved by the Research Ethics Committee of Universidade Nove de Julho (approval number 38816114.3.0000.5511) and carried out at the Physical Therapy Clinic of Universidade Nove de Julho. Written informed consent was obtained from parents or legal guardians, and assent was obtained from all participating children prior to enrollment [21].

### 2.2 Participants

Children aged 6 to 12 years with a medical diagnosis of asthma were recruited from the outpatient department at the Physical Therapy Clinic of Universidade Nove de Julho. Eligibility criteria included clinical stability at the time of assessment and the ability to perform spirometry and field exercise testing. Children were excluded if they had experienced an acute respiratory infection or asthma exacerbation within the preceding 2 months, had comorbid cardiovascular, neurological, musculoskeletal, or cognitive conditions that could interfere with test performance, or were unable to complete any of the study procedures [1, 6, 22].

### 2.3 Clinical and Anthropometric Assessment

Demographic data (age and sex) and anthropometric measurements were obtained for all participants. Body weight was measured using a calibrated digital scale, and height was measured with a wall‐mounted stadiometer. Body mass index (BMI) was calculated as weight divided by height squared (kg/m^2^), according to standard procedures for pediatric populations [23]. In addition, BMI *z*‐scores were calculated using the WHO 2007 BMI‐for‐age reference standards for children and adolescents aged 5–19 years [24]. Absolute BMI values were retained in analyses involving ISWT percentage predicted because the pediatric ISWT reference equation also incorporates BMI expressed in kg/m^2^.

### 2.4 Lung Function

Spirometry was performed using a calibrated spirometer following American Thoracic Society and European Respiratory Society guidelines [25]. Forced vital capacity (FVC), forced expiratory volume in 1 s (FEV_1_), FEV_1_/FVC ratio, and forced expiratory flow between 25% and 75% of FVC (FEF25–75%) were recorded. Results were expressed as percentages of predicted values based on age, sex, and height using reference equations appropriate for children [26]. Only technically acceptable and reproducible maneuvers were included in the analysis.

### 2.5 Functional Exercise Capacity

Functional exercise capacity was assessed using the Incremental Shuttle Walk Test (ISWT), performed on a 10‐m course with standardized audio signals to control walking speed [11]. Participants were instructed to walk back and forth between two cones at increasing speeds dictated by the audio recording. Standardized verbal encouragement was provided at the end of each level, in accordance with published recommendations for field walking tests [12]. Heart rate and peripheral oxygen saturation were monitored throughout the test. The test was terminated when the participant was unable to maintain the required pace or experienced limiting symptoms. The total distance walked was recorded and expressed as both absolute distance in meters and percentage of predicted distance. Percentage predicted values were calculated using the pediatric Brazilian reference equation proposed by Lanza et al., which was developed in healthy children and adolescents aged 6–18 years and includes age, sex, and body mass index as predictor variables [13]. This equation was selected because it was derived from a Brazilian pediatric population and is currently one of the few available reference equations for ISWT performance in children and adolescents. However, it has not been independently validated in the present sample of children with asthma.

### 2.6 Asthma Control and Quality of Life

Asthma control was assessed using the six‐item Asthma Control Questionnaire (ACQ‐6), a validated instrument for evaluating clinical control in asthma [27]. Quality of life was evaluated using the Pediatric Asthma Quality of Life Questionnaire (PAQLQ), which assesses symptoms, activity limitation, and emotional function and has been validated for use in pediatric populations [28, 29].

### 2.7 ICF‐Based Classification of Functional Limitation

Functional exercise capacity was interpreted using an exploratory operational framework conceptually aligned with the generic activity qualifiers of the International Classification of Functioning, Disability and Health (ICF). This approach was not intended to represent a formally validated ICF classification system, but rather to facilitate the clinical interpretation of ISWT performance within the activity domain of the ICF.

Because ISWT performance was expressed as a percentage of predicted distance, the ICF percentage ranges were applied inversely and symmetrically: higher percentages of predicted ISWT distance were interpreted as lower activity limitation, whereas lower percentages were interpreted as greater activity limitation. Thus, the proposed categories were conceptually aligned with the ordinal structure of ICF qualifiers, ranging from no limitation to complete limitation. Participants were grouped into mild/moderate or severe activity limitation categories for analytical purposes (Table 1) [18, 19].

**Table 1:** Exploratory operational alignment between generic ICF activity qualifiers and ISWT performance categories.

ICF qualifier	Qualitative description	ISWT (% predicted)
.0	No problem (0–4%)	96–100%
.1	Mild problem (5–24%)	76–95%
.2	Moderate problem (25–49%)	51–75%
.3	Severe problem (50–95%)	5–50%
.4	Complete problem (96–100%)	0–4%

*Note:* The proposed ISWT categories were conceptually inspired by the percentage structure of generic ICF qualifiers. They do not represent formally validated cutoffs for ISWT interpretation and should be considered an exploratory operational framework.

### 2.8 Statistical Analysis

Data distribution was assessed using the Shapiro–Wilk test [30]. Continuous variables were expressed as mean ± standard deviation or median and interquartile range, as appropriate. Group comparisons between children with mild/moderate and severe activity limitation were performed using independent t‐tests or Mann–Whitney U tests for continuous variables and chi‐square or Fisher's exact tests for categorical variables [31]. Effect sizes were calculated using Cohen's d or Hedges’ g to support clinical interpretation of group differences [32].

Associations between functional exercise capacity (ISWT % predicted) and clinical variables were examined using Pearson or Spearman correlation coefficients, as appropriate [33]. Multivariable linear regression analysis was conducted with ISWT percentage of predicted distance as the dependent variable, including age, sex, BMI, and FEV_1_ % predicted as independent variables, following standard recommendations for regression modeling in clinical research [34]. Because ISWT percentage predicted values were derived from a reference equation that includes age, sex, and body mass index, regression models including these variables were interpreted cautiously due to the potential for mathematical coupling. Therefore, multivariable analyses were considered exploratory and were not intended to establish causal or independent physiological determinants of ISWT performance. Statistical significance was set at *p* < 0.05. All analyses were performed using the appropriate statistical software, Minitab 14.

### 2.9 Sample Size and Power Considerations

The sample size was defined by feasibility, based on the number of eligible children who met the inclusion criteria during the study period. Given a total sample of 39 participants and a two‐sided significance level of 0.05, the study had approximately 80% statistical power to detect moderate‐to‐large associations between functional exercise capacity, expressed as ISWT percentage of predicted distance, and clinical or demographic variables, corresponding to correlation coefficients of approximately |*r* | ≥ 0.44.

For group‐based analyses derived from the ICF‐aligned ISWT classification, considering the observed group sizes (mild/moderate activity limitation, *n* = 11; severe activity limitation, *n* = 28), the study was sufficiently powered (80%) to detect large standardized mean differences between groups (Cohen's *d* ≥ 1.0). Therefore, the sample size was adequate to identify clinically meaningful differences in functional exercise capacity and to demonstrate dissociation between functional performance and resting lung function.

Smaller effect sizes in secondary outcomes, such as spirometric indices, asthma control, or quality of life measures, may not have been detectable with the present sample and were interpreted accordingly.

## 3 Results

### 3.1 Participant Characteristics

Thirty‐nine children with asthma were included in the analysis, of whom 23 (59%) were boys. The mean age was 8.6 ± 2.3 years. Anthropometric characteristics indicated a predominantly eutrophic sample. Resting lung function was relatively preserved, with mean forced vital capacity (FVC) of 95.5 ± 14.9% predicted and mean forced expiratory volume in 1 s (FEV_1_) of 82.0 ± 16.4% predicted.

In contrast, functional exercise capacity assessed by the Incremental Shuttle Walk Test (ISWT) was markedly reduced. The mean distance walked was 541.1 ± 240.7 m, corresponding to 41.4 ± 18.7% of the predicted distance. Detailed demographic, anthropometric, clinical, spirometric, and functional characteristics of the overall sample and according to functional limitation groups are presented in Table 2.

**Table 2:** Demographic, clinical, spirometric, and functional characteristics of participants according to ICF–ISWT classification.

Variable	All participants (*n* = 39)	Mild/Moderate (*n* = 11)	Severe (*n* = 28)
Sex – Female, *n* (%)	16 (41.0)	4 (36.4)	12 (42.9)
Sex – Male, *n* (%)	23 (59.0)	7 (63.6)	16 (57.1)
Age (years), median [IQR]	8.0 [7.0–10.5]	7.0 [6.0–8.0]	8.5 [7.0–11.0]
Weight (kg), median [IQR]	27.0 [22.6–39.5]	23.0 [20.0–31.0]	31.0 [24.6–40.1]
Height (m), mean (SD)	1.30 (0.18)	1.24 (0.19)	1.32 (0.17)
BMI (kg/m^2^), median [IQR]	17.36 [15.44–20.13]	19.46 [15.11–20.00]	17.03 [15.53–20.86]
BMI *z*‐score, median [IQR]	0.60 [−0.16 to 1.74]	1.11 [−0.21 to 1.94]	0.59 [−0.02 to 1.39]
ACQ‐6, median [IQR]	0.66 [0.16–1.83]	0.50 [0.24–1.24]	0.66 [0.16–2.54]
FVC (% predicted), mean (SD)	95.46 (14.90)	94.73 (8.44)	95.75 (16.90)
FEV_1_ (% predicted), mean (SD)	81.97 (16.39)	82.45 (14.01)	81.79 (17.47)
FEV_1_/FVC (% predicted), median [IQR]	83.33 [70.35–85.10]	83.64 [71.55–85.70]	83.21 [70.77–85.00]
FEF25–75% (% predicted), mean (SD)	68.18 (25.76)	68.27 (25.78)	68.14 (26.23)
ISWT distance (m), mean (SD)	541.10 (240.72)	840.64 (159.01)	423.43 (145.87)*
ISWT (% predicted), mean (SD)	41.41 (18.73)	65.26 (10.06)	32.04 (11.54)
PAQLQ – Symptoms, median [IQR]	65.0 [46.5–70.0]	70.0 [49.0–70.0]	65.0 [44.8–70.0]
PAQLQ – Emotions, median [IQR]	46.0 [33.0–52.0]	44.0 [33.5–48.5]	49.0 [33.0–52.0]
PAQLQ – Activities, mean (SD)	18.10 (8.57)	21.36 (8.29)	16.82 (8.48)
PAQLQ – Total, median [IQR]	126.0 [105.0–137.5]	123.0 [109.0–130.0]	126.0 [100.0–138.0]

Abbreviations: ACQ‐6, Asthma Control Questionnaire 6; BMI, Body mass index; FEF25–75%, Forced expiratory flow between 25% and 75% of FVC; FEV1, Forced expiratory volume in one second; FVC, Forced vital capacity; ICF, International Classification of Functioning, Disability and Health; IQR, Interquartile Range; ISWT, Incremental Shuttle Walk Test; PAQLQ, Pediatric Asthma Quality of Life Questionnaire; SD, Standard deviation. BMI *z*‐score was calculated according to the WHO 2007 BMI‐for‐age reference for children and adolescents aged 5–19 years.

### 3.2 CF‐Based Classification of Functional Exercise Capacity

Based on the classification presented in Table 1, 11 children (28%) were categorized as having mild/moderate activity limitation (ICF qualifiers 0.1–0.2; ISWT 51–95% predicted), whereas 28 children (72%) were classified as having severe activity limitation (ICF qualifiers 0.3–0.4; ISWT ≤ 50% predicted).

### 3.3 Comparison Between Functional Limitation Groups

Children classified as having mild/moderate activity limitation demonstrated substantially higher functional exercise capacity compared with those classified as having severe limitation. The mild/moderate group walked a mean distance of 840.6 ± 159.0 m (65.3 ± 10.1% predicted), whereas the severe group walked 423.4 ± 145.9 m (32.0 ± 11.5% predicted; *p* < 0.001), corresponding to a very large effect size (Cohen's *d* = 2.79).

In contrast, resting lung function did not differ between functional categories. Mean FVC was 94.7 ± 8.4% predicted in the mild/moderate group and 95.8 ± 16.9% predicted in the severe group (*p* = 0.80). Mean FEV_1_ was 82.5 ± 14.0% predicted and 81.8 ± 17.5% predicted in the mild/moderate and severe groups, respectively (*p* = 0.90). No significant between‐group differences were observed for FEV_1_/FVC ratio or FEF25–75%.

Clinical variables, including asthma control assessed by ACQ‐6, resting and peak heart rate, peripheral oxygen saturation during the ISWT, and Pediatric Asthma Quality of Life Questionnaire (PAQLQ) total and domain scores, were also similar between groups (all *p* > 0.05). Full between‐group comparisons are shown in Table 2.

### 3.4 Associations With Functional Exercise Capacity

Correlation analyses between ISWT percentage of predicted distance and clinical variables revealed no significant associations with spirometric indices, asthma control, or quality of life measures. However, ISWT % predicted showed a significant negative association with age, indicating lower relative functional performance with increasing age. Correlation coefficients are summarized in Table 3.

**Table 3:** Correlations between ISWT % predicted and clinical variables.

Variable	Correlation coefficient (*r*)	*p*‐value
Age (years)	–0.40	0.01*
BMI (kg/m^2^)	–0.08	ns
FEV_1_ (% predicted)	0.04	ns
FVC (% predicted)	–0.03	ns
FEF25–75% (% predicted)	–0.02	ns
ACQ‐6	–0.12	ns
PAQLQ – Total	0.09	ns

Abbreviations: ACQ‐6, Asthma Control Questionnaire 6; BMI, Body mass index; FEF25–75%, Forced expiratory flow between 25% and 75% of FVC; FEV1, Forced expiratory volume in one second; FVC, Forced vital capacity; ISWT, Incremental Shuttle Walk Test; PAQLQ, Pediatric Asthma Quality of Life Questionnaire.

* *p* = 0.05.

### 3.5 Multivariable Analysis

Multivariable linear regression analysis was performed with ISWT percentage of predicted distance as the dependent variable. After adjustment for sex, body mass index, and FEV_1_ % predicted, age emerged as the only independent predictor of functional exercise capacity. Lung function and asthma control variables were not independent predictors in the model. Results of the regression analysis are presented in Table 4.

**Table 4:** Multivariable linear regression analysis with ISWT % predicted as the dependent variable.

Predictor	β coefficient	95% CI	*p*‐value
Age (years)	–3.47	–6.25 to –0.69	0.016*
Sex (male)	2.10	–6.40 to 10.60	0.62
BMI (kg/m^2^)	–0.85	–2.10 to 0.40	0.18
FEV_1_ (% predicted)	0.09	–0.15 to 0.33	0.46

*Note:* Forced expiratory volume in one second (FEV_1_); Model fit: *R* ^2^ = 0.18; adjusted *R* ^2^ = 0.08.

Abbreviations: BMI, body mass index; ISWT, Incremental Shuttle Walk Test.

* *p* < 0.05.

**Figure 1:**
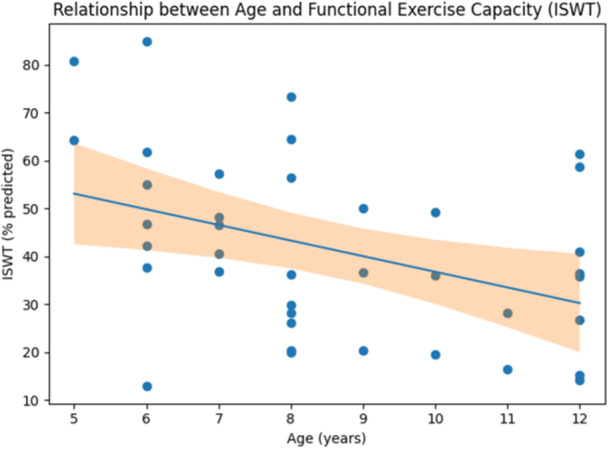
Relationship between age and functional exercise capacity is expressed as the Incremental Shuttle Walk Test (ISWT) percentage of predicted distance. Each point represents an individual participant. The solid line represents the linear regression, and the shaded area indicates the 95% confidence interval. [Color figure can be viewed at wileyonlinelibrary.com]

As shown in Figure 1, ISWT performance expressed as a percentage of predicted distance demonstrated a negative association with age. However, because age is incorporated into the reference equation used to derive ISWT percentage predicted values, this finding should be interpreted cautiously due to the potential influence of mathematical coupling.

**Figure 2:**
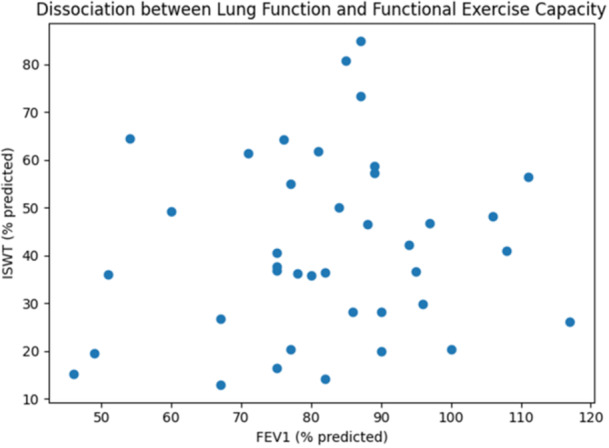
Relationship between forced expiratory volume in one second (FEV_1_, % predicted) and functional exercise capacity expressed as Incremental Shuttle Walk Test (ISWT) percentage of predicted distance. Each point represents an individual participant. [Color figure can be viewed at wileyonlinelibrary.com]

As illustrated in Figure 2, no clear relationship was observed between resting lung function, assessed by FEV_1_ (% predicted), and functional exercise capacity measured by ISWT performance, supporting the dissociation between spirometric indices at rest and exercise‐based functional limitation.

## 4 Discussion

The present study demonstrates that children with asthma may exhibit substantial functional exercise limitation when assessed by the Incremental Shuttle Walk Test (ISWT), despite relatively preserved lung function at rest and similar levels of asthma control and quality of life. By operationally aligning ISWT performance with generic International Classification of Functioning, Disability and Health (ICF) activity qualifiers, this study highlights a dimension of functional impairment that is not captured by conventional clinical assessments, reinforcing the dissociation between resting pulmonary function and functional capacity during exercise.

One of the main findings of this study is the marked discrepancy between spirometric indices and functional exercise performance. Although mean FEV_1_ and FVC values were within expected ranges, most participants demonstrated severely reduced ISWT performance, with more than 70% classified as having severe activity limitation according to the ICF‐based classification. This finding is consistent with previous studies showing that resting lung function poorly reflects exercise tolerance and functional status in children with asthma [7, 8, 9, 10]. Spirometry assesses airflow limitation under static conditions and does not account for dynamic ventilatory constraints, cardiovascular responses, peripheral muscle function, or behavioral adaptations that may limit exercise performance during physical activity [10].

These findings reinforce that functional exercise capacity represents an integrated outcome of multiple physiological systems and contextual factors. Consequently, reliance solely on spirometric measurements may underestimate the real‐life functional impact of asthma, particularly in children who appear clinically stable [9, 10].

Field‐based exercise tests, such as the ISWT, provide a practical and clinically meaningful method for evaluating functional exercise capacity in pediatric populations [11, 12]. The ISWT is externally paced and incremental, closely reflecting the physiological demands of daily physical activities. In the present study, ISWT performance clearly discriminated between levels of functional limitation, yielding a very large effect size between mild/moderate and severe activity limitation groups, while spirometric and clinical variables failed to do so.

Previous studies have demonstrated reduced walking distance and impaired exercise tolerance in children with asthma when assessed by field tests, even in those with preserved lung function [7, 8, 9]. Expressing ISWT performance as a percentage of predicted values allowed comparison across ages and body sizes and facilitated its clinical interpretation [13]. These findings support the potential usefulness of the ISWT as a complementary functional assessment tool in pediatric asthma assessment.

It should also be noted that currently available pediatric ISWT reference equations have important limitations. The equation used in the present study was developed in healthy Brazilian children and adolescents and explained approximately 48% of the variance in ISWT distance. In addition, the equation does not provide residual standard error, standard deviation of residuals, or other parameters required to calculate z‐scores or lower limits of normality. Therefore, although percentage predicted values allow relative comparison across age and anthropometric characteristics, they should be interpreted cautiously and do not replace LLN‐ or z‐score‐based interpretation when such parameters become available.

The integration of functional exercise performance with the ICF framework represents a clinically relevant approach to interpreting activity limitations in pediatric asthma. Rather than serving as a diagnostic classification system, the ICF provides a biopsychosocial model that facilitates the interpretation of functional outcomes across different health domains. In this context, the exploratory operational alignment between ISWT performance and generic ICF activity qualifiers was intended to contextualize objective exercise limitations within the activity domain, complementing symptom‐based and quality of life assessments.

Importantly, the ICF does not provide exercise test‐specific quantitative thresholds. Therefore, the proposed ISWT categories should not be interpreted as formally validated ICF classifications, but rather as a conceptual and exploratory framework inspired by the ordinal structure and percentage ranges of the generic ICF qualifiers. This functioning‐oriented perspective may facilitate interdisciplinary communication and support a more comprehensive description of functional limitations in children with asthma, consistent with previous applications of the ICF in pediatric respiratory conditions [18, 19, 35].

An additional finding of this study was the negative association between age and ISWT performance expressed as a percentage of predicted distance. However, this finding should be interpreted cautiously because age is incorporated into the reference equation used to calculate ISWT percentage‐predicted values. Therefore, the observed association may partially reflect mathematical coupling related to the structure of the prediction equation rather than a true physiological decline in functional exercise capacity.

Although older children demonstrated lower relative ISWT performance, the cross‐sectional design of this study does not allow causal inference regarding mechanisms underlying this finding. Hypothetical explanations may include differences in habitual physical activity, exercise participation, or behavioral adaptations over time; however, these variables were not directly measured and require confirmation in future longitudinal studies incorporating objective assessments of physical activity and physical conditioning [8, 14].

Because predicted ISWT values increase with age, failure to achieve age‐appropriate performance may reflect a growing gap between expected and actual functional capacity [14, 15]. This highlights the importance of early identification of functional limitations and timely interventions aimed at promoting physical activity and exercise participation.

The absence of significant associations between functional exercise capacity and asthma‐specific quality of life measures in the present study deserves careful interpretation. Previous work has demonstrated that widely used pediatric asthma quality of life instruments, including the PAQLQ, predominantly address domains related to body functions, with more limited coverage of activity and participation components when analyzed within the ICF framework. As shown by Gomes et al. [20], the PAQLQ contains a higher concentration of items linked to physiological and emotional functions, whereas fewer items reflect mobility, physical activities, or participation in daily life. Consequently, objective limitations in functional exercise capacity may not be adequately captured by patient‐reported quality of life measures, particularly in children who adapt their activities to avoid symptoms [5, 9].

Nevertheless, previous studies have shown that sleep disturbances, asthma severity, and physical inactivity can negatively affect both quality of life and exercise capacity [5, 16, 17]. The lack of association observed in the present study may therefore reflect limited statistical power to detect small‐to‐moderate effects, differences in sample characteristics, or adaptive behavioral strategies adopted by children with asthma.

This study has several strengths, including the use of a validated field exercise test, the integration of functional performance with the International Classification of Functioning, Disability and Health (ICF) framework, and the focus on clinically meaningful outcomes. However, some limitations should be acknowledged. The cross‐sectional design precludes causal inference, and the relatively small sample size may have limited the detection of small‐to‐moderate associations in secondary outcomes. Additionally, objective measures of daily physical activity and sedentary behavior were not included, which may have provided further insight into the mechanisms underlying functional limitation [14].

Furthermore, the ICF‐aligned ISWT categories proposed in this study have not been formally validated. These categories were developed as an exploratory operational approach conceptually inspired by the percentage structure of generic ICF qualifiers and should not be interpreted as validated diagnostic thresholds. Another limitation is that ISWT z‐scores and lower limits of normality could not be calculated because the pediatric reference equation used does not provide the residual variability parameters required for these estimates. Finally, because ISWT performance was expressed as a percentage of predicted distance, associations involving variables incorporated into the reference equation, particularly age, sex, and BMI, should be interpreted cautiously due to potential mathematical coupling.

Future studies should validate these exploratory categories against clinical outcomes, objective physical activity measures, and responsiveness to rehabilitation interventions.

The findings of this study have important clinical implications. Functional exercise limitations may be present even in children with apparently well‐controlled asthma and preserved lung function. Incorporating functional assessments such as the ISWT, interpreted within the ICF framework, may help identify children who could benefit from targeted interventions focused on physical conditioning, exercise training, and activity promotion [15].

Future research should explore longitudinal changes in functional capacity, validate ICF‐aligned ISWT categories against external clinical outcomes, and incorporate objective measures of daily physical activity, sedentary behavior, and physical conditioning. In addition, intervention studies are needed to assess the responsiveness of this exploratory framework to exercise‐based and rehabilitation programs in children with asthma.

In conclusion, children with asthma may present substantial limitations in functional exercise capacity despite relatively preserved lung function and similar levels of asthma control and quality of life. These findings reinforce the dissociation between resting pulmonary function and exercise‐based functional performance and highlight the limitations of conventional clinical assessments in capturing activity‐related impairment.

The exploratory operational alignment of Incremental Shuttle Walk Test performance with generic International Classification of Functioning, Disability and Health activity qualifiers may provide a clinically useful framework to support interpretation of functional exercise limitations within the activity domain. However, this approach should be interpreted cautiously and requires future validation before broader clinical application.

Incorporating objective functional assessments such as the ISWT into the clinical evaluation of pediatric asthma may support a more comprehensive understanding of disease impact and help guide targeted interventions aimed at improving physical conditioning, exercise participation, and functional capacity.

## Author Contributions

**Danilo Rufino Cavalcante de Souza:** investigation, writing – original draft, methodology, visualization, writing – review and editing, formal analysis, data curation. **Ivan Peres Costa:** conceptualization, formal analysis, writing – review and editing, validation, methodology, supervision. **Etiene Farah Teixeira de Carvalho:** investigation, writing – original draft, methodology, validation, writing – review and editing, data curation, supervision. **Dirceu Costa:** conceptualization, investigation, methodology, validation, visualization, writing – review and editing, data curation, supervision. **Evelim Leal Freitas Dantas Gomes:** conceptualization, investigation; writing – original draft, methodology, validation, visualization, writing – review and editing, formal analysis, project administration, data curation, supervision.

## Funding

The authors have nothing to report.

## Conflicts of Interest

The authors declare no conflicts of interest.

## Data Availability

The data that support the findings of this study are available from the corresponding author upon reasonable request.
